# Altered iron metabolism and iron therapy in restless legs syndrome

**DOI:** 10.1007/s00702-026-03274-1

**Published:** 2026-09-24

**Authors:** Zhikui Wei, Chrsitopher J. Earley

**Affiliations:** 1 Department of Neurology, Department of Psychiatry & Human Behavior, Jefferson Sleep Disorders Center, Thomas Jefferson University Hospital, Philadelphia, PA USA; 2 Center for Restless Legs Study, Department of Neurology, Johns Hopkins University, Baltimore, MD USA

**Keywords:** Restless legs syndrome, Iron metabolism, Iron therapy, Brain iron deficiency

## Abstract

Restless legs syndrome (RLS) is a common neurological disorder that impairs sleep and reduces quality of life. The pathogenesis of RLS is incompletely understood. However, two mechanisms in its pathogenesis have been well accepted: iron deficiency, particularly brain iron deficiency, and subsequent dopamine dysregulation, which contribute to sensorimotor symptoms in RLS. In this review, we aim to provide a balanced synthesis of the literature on peripheral and brain iron dysregulation and iron therapy in RLS. We conclude that brain iron deficiency is central to the pathogenesis of RLS, whereas peripheral iron deficiency, although closely linked to RLS, poorly reflects brain iron status. Brain iron deficiency in RLS likely arises from altered iron metabolism at multiple levels, including impaired iron transport at the blood-brain interface and in subcortical neuronal structures such as the dopaminergic and thalamic systems, and potentially altered iron export, with or without systemic iron deficiency. These processes are further modulated by circadian-related changes, genetic susceptibility, and sex differences, adding complexity to iron dysregulation in RLS. Iron therapy, both oral and IV formulations, has become a standard of care in the treatment of RLS. Further research is needed to deepen our understanding of brain iron deficiency, improve the prediction of treatment response, and optimize the selection of iron therapies for RLS.

## Introduction

Restless legs syndrome (RLS) is characterized by severe discomfort primarily in the legs, accompanied by the urge to move (Allen et al. 2003). Neurologically, this can be conceptualized as a type of akathisia (Earley 2003). Unlike other forms of akathisia, such as neuroleptic-induced akathisia, RLS exhibits a distinct circadian pattern, with symptoms starting or worsening at night or around bedtime (Ferré et al. 2024). Other defining clinical features of RLS include symptom onset during periods of rest or inactivity, particularly when attempting to sleep. In contrast to neuroleptic-induced akathisia, RLS symptoms should be fully relieved by prolonged motor activity (e.g., walking) or sustained mental engagement (Allen et al. 2003).

RLS occurs in 3.9–14.3% of the general population, more commonly in women, and the incidence increases with age (Ohayon et al. 2012). Certain populations demonstrate substantially higher prevalence of RLS, such as pregnant women, individuals with iron deficiency anemia (IDA), and patients with chronic kidney disease (CKD), with reported rates of approximately 25% during pregnancy (Lepuzanović et al. 2025), 35–50% in IDA (Allen et al. 2013a), and 15–30% in CKD (Safarpour et al. 2023). RLS is associated with a significant decrease in quality of life (Abetz et al. 2004), underscoring the importance of careful evaluation and treatment.

The pathogenesis of RLS is complex and incompletely understood. Currently, two pathogenic mechanisms are well accepted: iron deficiency, particularly brain iron deficiency, and dopamine dysregulation (Earley et al. 2014). It is thought that brain iron deficiency or cellular iron insufficiency in certain brain regions leads to altered neurochemical responses in the dopamine system, including dopamine synthesis, transport, and receptor activity, resulting in the activation of sensorimotor symptoms. Brain iron deficiency has been associated with increased tyrosine hydroxylase activity (Connor et al. 2009), a key enzyme involved in dopamine synthesis, possibly increased extracellular dopamine (Nelson et al. 1997), decreased membrane-bound dopamine transporter (DAT) (Earley et al. 2011; Lin et al. 2016), and reduced expression of dopamine receptor D2R in the putamen (Connor et al. 2009). Additionally, brain iron deficiency may alter other neurochemical pathways, such as the glutamatergic (Allen et al. 2013b) and adenosinergic systems (Ferre et al. 2017), which also contribute to the sensorimotor symptoms of RLS.

This review aims to provide an updated and balanced perspective on iron metabolism and iron therapy in RLS. We place particular emphasis on integrating current knowledge of systemic iron metabolism relevant to RLS (Table 1) with mechanisms more specific to the disorder, such as iron transport across the blood-brain barrier, circadian regulation of iron metabolism, disease-associated genetic variants, and brain iron deficiency (Table 2). Although numerous mechanistic studies have been conducted in animal models, such as diet-induced brain iron deficiency rodent models (Earley et al. 2022), the present review primarily focuses on human clinical and epidemiologic studies. It includes animal and cell culture studies when necessary to illustrate mechanistic insights into the dysregulation of iron metabolism in RLS. We start with a basic introduction to iron biology relevant to RLS (Table 1), followed by a synthesis of the literature across several domains of iron metabolism in RLS, including epidemiology, neurochemistry, neuropathology, genetics, and an up-to-date synthesis of iron therapy.

**Table 1:** Summary of Iron Metabolism and RLS Correlates

Iron Metabolism	Cellular/Molecular Targets	Functions	RLS Correlates
Iron Absorption	Ferrireductases	Reduce Fe^3+^ to Fe^2+^	
	DMT1	Transports Fe^2+^ from duodenal lumen to enterocytes & from endosomes to cytoplasm	SN DMT1↓
	Hepcidin	Degrades ferroportin; reduces iron absorption and export	SN & putamen pro-hepcidin↑
Iron Export	Ferroportin	Cellular iron exporter	SN Ferroportin↓ Peripheral (lymphocytes, nEVs): Ferroportin↑
Iron Uptake (across BBB)	Transferrin	Primary iron carrier protein	Peripheral (subset) transferrin↑ CSF transferrin↑ SN transferrin↑
	TfR1	Primary transferrin receptor (iron uptake)	SN TfR1↓
	(Secreted) Ferritin	Iron carrier protein	Peripheral (subset) ferritin ↓ CSF ferritin↓
	TfR1, Tim-1/2, Scara5, CXC4	Ferritin receptors	
Iron Storage	Intracellular ferritin	Sequesters iron intracellularly	SN H-ferritin↓
Iron Utilization	Heme biosynthesis; iron-dependent enzymes	Erythropoiesis; iron-dependent metabolic reactions (e.g., mitochondrial respiration, neurotransmitter synthesis, myelination)	Striatum dopamine synthesis↑
Iron Recycling	Macrophages	Erythrophagocytosis, ferroportin-mediated export, ferritin secretion	

*BBB* blood-brain barrier, *CSF* cerebrospinal fluid, *CXCR4* C-X-C chemokine receptor type 4, *DMT1* divalent metal transporter 1, *Fe*²⁺ ferrous iron; *Fe*³⁺ ferric iron, *H-ferritin* heavy-chain ferritin, *nEVs* neuron-derived extracellular vesicles, *RLS* restless legs syndrome, *SN* substantia nigra; *TfR1* transferrin receptor 1, *Tim-1/2* T-cell immunoglobulin and mucin domain proteins 1 and 2

**Table 2:** Summary of Studies on Brain Iron Deficiency in RLS

Investigations	Major Findings	Conclusions
CSF Studies	↓ CSF ferritin, ↑ CSF transferrin (normal serum levels) in RLS Serum–CSF ferritin correlation present but weaker in RLS Earlier RLS onset associated with lower CSF ferritin ↓ H- and L-ferritin in early-onset RLS (< 45 year)	• Evidence of overall decreased brain iron store in RLS • More prominent brain iron deficiency in early onset RLS
Brain MRIs	↓ iron content in SN; mild reduction in putamen ↓ iron index in SN correlated with symptom severity in early-onset RLS Brain iron indices across multiple regions show heterogeneous results Brain iron indices increase after IV iron therapy and correlate with symptom improvement	Impaired brain iron homeostasis in RLS particularly in SN Sensorimotor network (dopaminergic-thalamic pathways) likely involved in pathophysiology and treatment response
TCUS	SN hypoechogenicity in RLS SNEI increases after IV iron therapy	Reduced SN iron content in RLS SNEI may predict therapeutic response to iron therapy
Neuropathology	↓ iron staining, H-ferritin, DMT1, ferroportin, and TfR, and ↑ transferrin in SN in RLS L-ferritin redistributed to astrocytic-type cells ↑ mitochondrial ferritin in SN neurons Abnormal iron handling at blood-brain interface (microvessels, choroid plexus)	Cellular iron insufficiency in SN and related regions in RLS Dysregulated neuronal iron acquisition in SN in RLS Impaired iron transport across the blood-brain interface in RLS
Studies on Iron Export	↑ ferritin (mainly H-ferritin) in nEV in RLS ↑ ferritin and ferroportin, ↓ TfR in nEVs from iron-deficient RLS patients ↑ ferroportin, DMT1, and TfR in lymphocytes in RLS Faster systemic iron loss after IV iron infusion	Possible increased cellular/neural iron export and systemic iron elimination in RLS Altered iron homeostasis in RLS may extend beyond the brain

*CSF* cerebrospinal fluid, *DMT1* divalent metal transporter 1, *nEV* neuronal extracellular vesicle, *SN* substantia nigra, *SNEI* substantia nigra echogenicity index, *TCUS* transcranial ultrasound, *TfR* transferrin receptor

## Basic iron biology

A brief overview of general iron metabolism is provided to facilitate the subsequent discussion of altered iron metabolism in RLS (Tables 1 and 2). Readers seeking a more comprehensive overview of systemic iron metabolism are referred to the following reviews (Pantopoulos et al. 2012; Ganz 2013).

### Iron absorption

Iron exists primarily in two ionic forms: ferrous (Fe²⁺) and ferric (Fe³⁺) iron (Pantopoulos et al. 2012). Iron in the diet mainly exists in heme, ferritin, and ferric ions complexed with other molecules (Ganz 2013). Absorption of ferric iron starts with reduction to ferrous iron by ferrireductases such as duodenal cytochrome B, often with vitamin C as a cofactor (McKie et al. 2001). Once reduced, ferrous iron enters enterocytes via the divalent metal transporter-1 (DMT1) transporter (Pantopoulos et al. 2012). In contrast, despite the nutritional importance of heme iron, molecular pathways for the absorption of dietary heme and ferritin iron are less well defined (Pantopoulos et al. 2012; Chang et al. 2023; Lee et al. 2025). Intestinal heme absorption is currently proposed to involve a heme transporter or receptor, although the identity of this transporter/receptor remains controversial (Lee et al. 2025). Inside enterocytes, some iron is stored, while the rest is exported across the basolateral membrane by ferroportin, the primary cellular iron exporter (Pantopoulos et al. 2012). The peptide hormone hepcidin, produced by the liver, regulates this process by binding to ferroportin, causing its internalization and degradation, thereby reducing iron absorption (Pantopoulos et al. 2012).

### Iron transport, storage, and uptake

The export of iron from enterocytes is coupled with iron oxidation by hephaestin and ceruloplasmin, through which ferrous iron is converted back to ferric iron (Pantopoulos et al. 2012). Ferric iron is then bound by transferrin, a key carrier protein that maintains iron in a soluble, nonreactive state in circulation. Most transferrin-bound iron is delivered to the bone marrow for red blood cell production. Other iron is transported to the liver for long-term storage and to various tissues and organs to participate in biological reactions or to be stored within cells. Ferritin plays a central role in safely sequestering iron within cells. Ferritin is a 24-subunit heteropolymer made of varying ratios of H- and L-ferritin. H-ferritin, which is more metabolically active, catalyzes the oxidation of ferrous iron to ferric iron and stores iron short-term. At the same time, L-ferritin promotes nucleation and long-term storage of ferric iron within the ferritin core (Wade, Chiou, and Connor 2019). While most ferritin remains intracellular, a small portion is released into the serum. Serum ferritin is a widely used biomarker of iron stores in the body (Andrews 2008).

Transported iron must cross the blood-brain barrier to enter the brain. Two major forms of iron uptake across the blood-brain barrier have been described: transferrin-bound iron and ferritin-bound iron (Wade, Chiou, and Connor 2019). Transferrin-mediated iron uptake represents the classical pathway for iron delivery and is described as a slow, steady system that supplies iron to most cell types in the body (Wade, Chiou, and Connor 2019). At the blood-brain barrier, iron-loaded transferrin binds to transferrin receptor 1 (TfR1) on endothelial cells and undergoes endocytosis (Roberts et al. 1993). Subsequently, iron is released from endosomes for intracellular use, storage, or export into the brain parenchyma (Wade, Chiou, and Connor 2019). In contrast, ferritin-mediated iron uptake across the blood-brain barrier is more complex and less understood. Although ferritin is primarily an intracellular iron storage protein, it is also secreted via secretory autophagy and exosomes (Truman-Rosentsvit et al. 2018). Secreted ferritin has a higher H- to L- ferritin ratio than serum ferritin and may facilitate more efficient iron delivery to target tissues. Ferritin interacts with multiple receptors, including TfR1, T-cell immunoglobulin and mucin domain receptor 1/2 (Tim-1/2), scavenger receptor class A member 5 (Scara5), and C-X-C chemokine receptor type 4 (CXCR4) (Wade, Chiou, and Connor 2019). Ferritin-mediated iron uptake via these receptors likely undergoes complex regulation and may function as a more efficient mechanism for iron delivery to the brain and other organs (Wade, Chiou, and Connor 2019; Fisher et al. 2007).

Compared with iron uptake at the BBB, i.e., iron in circulation passing through the luminal membrane of the BBB (blood side), how iron passes through the abluminal membrane (cerebral side) of the BBB and enters the brain remains less understood (Guo et al. 2026). Ferroportin was thought to be a key player in this process, coupled with hephaestin and ceruloplasmin, in a manner similar to iron transport across the basement membrane of enterocytes (Guo et al. 2026). Recent in vitro evidence suggests that iron transport across the BBB is also mediated by exosomes (Palsa et al. 2023), although the physiological importance of this pathway in vivo remains to be established.

### Iron utilization and recycling

Iron supports diverse biological functions by serving as a cofactor for enzymes involved in many essential processes, including cellular respiration, DNA synthesis, and gene regulation (Pantopoulos et al. 2012). Iron is particularly important in the brain, where it is required for myelin production and cellular respiration due to the brain’s disproportionately high oxygen consumption (Wade, Chiou, and Connor 2019). Iron is also necessary for the synthesis of several neurotransmitters, including dopamine, which plays a central role in RLS pathogenesis (Cooper, Bloom, and Roth 2003). Importantly, plasma iron is predominantly replenished by recycled iron from senescent red blood cells in the reticuloendothelial system (Pantopoulos et al. 2012). Macrophages play an essential role in this recycling process and, therefore, are the major source of the body’s day-to-day iron needs (Pantopoulos et al. 2012). Overall, iron homeostasis depends on the balance of absorption, utilization, storage, and recycling(Andrews 2008).

### Circadian regulation of iron metabolism

RLS is known to have a circadian pattern in symptom expression (Allen et al. 2003). Severity in idiopathic RLS is usually worse in the evening and at night, with the peak in the few hours after midnight, and better in the late morning (Allen et al. 2003). This is not entirely accounted for by reduced activity or sleep impairment at night but likely represents a true circadian variation in disease expression (Hening et al. 1999). For example, sensory discomfort and periodic leg movements in RLS patients showed a strong circadian variation in a modified 28-hour constant routine protocol, and have been linked to classic circadian biomarkers, such as salivary melatonin concentration and core body temperature (Michaud et al. 2004).

Iron metabolism exhibits circadian variation and may contribute to the circadian pattern of RLS. In humans, serum iron concentration follows a ~ 24-hour rhythm, peaking in the early morning and reaching a nadir around midnight (Kuhn and Brodan 1982). In primates, serum iron is higher in the morning and lower at night, whereas serum ferritin shows the opposite pattern: lower in the morning and higher at night. CSF iron and ferritin also exhibit circadian variation, with lower levels in the morning and higher levels later in the day (Hyacinthe et al. 2015). These findings may be relevant to RLS pathophysiology. It has been hypothesized that patients with RLS fail to show an adequate evening rise in CSF iron, potentially contributing to RLS onset in the evening or at night, supported by observations of lower nighttime CSF ferritin levels in patients with RLS compared with controls (Earley et al. 2005a, 2022). Interestingly, sleep deprivation decreases both the mean plasma iron levels and the circadian amplitude of plasma iron (Kuhn and Brodan 1982), suggesting that alterations in circadian regulation of iron may contribute to the exacerbation of RLS in the setting of sleep deprivation.

Animal studies focused on iron deficiency provided additional insights into the potential role of iron metabolism in circadian expression of RLS symptoms. In mice, iron deficiency alters diurnal regulation of dopaminergic systems (Bianco et al. 2009). More specifically, iron deficiency lowered dopamine transporter density in both the light and dark phases, but increased 3,4-dihydroxyphenylacetic acid (DOPAC)/dopamine ratio, a marker for dopamine turnover, only in the dark phase (Bianco et al. 2009). In addition, dietary iron deficiency reduced iron and ferritin levels in specific mouse brain regions, including the ventral midbrain and nucleus accumbens in both light and dark phase, and reduced whole brain iron levels only in the light phase (Unger et al. 2009). However, the diurnal variation in iron levels in brain regions such as the ventral midbrain appears to be strain- and sex-dependent, suggesting additional regulatory mechanisms of brain iron metabolism, including genetic background and sex (Unger et al. 2009, 2014).

## Peripheral iron deficiency and RLS

The Swedish neurologist Karl-Axel Ekbom, who first described RLS in detail in the 1940 s, noted its frequent occurrence during pregnancy and in iron-deficiency anemia (Ekbom and Ulfberg 2009). Menninger-Lerchental reported low blood iron levels in RLS in the 1960 s (Coccagna et al. 2004). O’Keeffe et al. (1994) found an inverse correlation between serum ferritin and RLS severity (rho=−0.53) in older adults. In 1997, Sun et al. also reported that serum ferritin was inversely correlated with RLS severity and positively correlated with sleep efficiency (rho = 0.48)(Sun et al. 1998). Since then, the association between iron deficiency and RLS has been replicated in diverse populations, including Indian (Rangarajan and D’Souza 2007), Turkish (Çurgunlu et al. 2012), and Korean cohorts (Bae et al. 2021a, b), as well as in children (Halac et al. 2015). It has also been demonstrated in conditions associated with iron deficiency, including iron deficiency anemia (Rangarajan and D’Souza 2007; Allen et al. 2013a; Kolukisa et al. 2016; Bae et al. 2021a, b), pregnancy (Lee et al. 2001; Minár et al. 2015; Telarović and Čondić 2019), frequent blood donations (Birgegård et al. 2010; Bryant et al. 2013), and polycythemia vera (Tobiasson et al. 2010). A lower ferritin level is also associated with augmentation of dopamine agonists in the treatment of RLS (Trenkwalder et al. 2008; Frauscher et al. 2009; Li et al. 2023).

In chronic kidney disease, another condition strongly associated with RLS, the association between iron deficiency and RLS remains incompletely established (Safarpour et al. 2023; Riar et al. 2019). Some studies have pointed to alternative or complementary mechanisms, such as inflammation (Higuchi et al. 2015), in addition to peripheral iron status (Elangovan et al. 2025). Important differences in patient characteristics, such as disease stage, age (pediatric vs. adult), and dialysis status, could also contribute to the heterogeneous findings across studies on iron and RLS in CKD. Similarly, in polycythemia vera, disease-related factors, such as gene mutations and cytoreductive treatment, have also been shown to affect the risk of RLS (Seyhan Erdoğan et al. 2023).

Of note, several studies found no association between RLS or RLS loci and peripheral iron deficiency in community-based samples (Hogl et al. 2005; Berger et al. 2002; Oexle et al. 2013; Lammers et al. 2019). Given that iron deficiency is less prevalent in community-based samples than in hospital- or clinic-based cohorts, this difference in baseline prevalence may help explain why studies in community samples fail to detect significant associations between iron deficiency and RLS. This hypothesis requires confirmation in future studies. Additionally, technical factors, such as the assays used for ferritin measurements (Majoni et al. 2023) and the time of day when iron labs were collected, given the circadian regulation of iron parameters (Bennett et al. 2024), may also influence results when evaluating peripheral iron status in patients with RLS. The current AASM guidelines recommend that testing of iron parameters should be performed in the morning with no iron-containing supplements or food for at least 24 h prior to the lab (Winkelman et al. 2025).

## Brain iron deficiency and RLS

Norlander postulated in 1953 that there could be tissue-specific iron deficiency in the presence of normal serum iron (Nordlander 1953). This hypothesis has been substantiated by the large body of research over the last 30 years, which has indeed demonstrated brain-specific iron deficiency in RLS. Brain iron deficiency (BID) has emerged as a core pathological mechanism in RLS. We will summarize evidence on BID in RLS, drawing from studies employing CSF biomarkers, neuroimaging techniques, and neuropathological analyses (Table 2).

### Alterations of CSF iron indices in RLS

Earley et al. first reported lower CSF ferritin and higher CSF transferrin levels in patients with RLS compared with controls, despite similar serum ferritin and transferrin levels between the two groups (Earley et al. 2000). This observation suggested reduced brain iron in RLS. A subsequent study showed a positive correlation between serum and CSF ferritin in both the control and RLS groups. However, the slope of the regression line in the CSF group was significantly lower (Mizuno et al. 2005). Later, CSF ferritin levels were found to correlate with the age of onset of RLS: the earlier the onset, the lower the CSF ferritin levels (Earley et al. 2005a). Moreover, both H- and L-ferritin were lower in the early-onset RLS group (< 45 years) but not in the late-onset RLS group (≥ 45 years) compared with the controls (Clardy et al. 2006a). Collectively, these studies provided evidence of overall decreased brain iron stores in RLS.

### Neuroimaging findings of brain iron deficiency in RLS

Neuroimaging has been extensively used to study brain iron metabolism in RLS. Allen et al. first used an MRI-based method and demonstrated decreased iron concentration in the substantia nigra (SN) and, to a lesser extent, in the putamen (Allen et al. 2001). Subsequent work showed that iron concentration in SN was decreased in early-onset RLS but not in late-onset RLS compared to controls, and the iron index in SN was correlated with the severity of RLS in patients with early-onset RLS (Earley et al. 2006). In addition, multiple MRI-based investigations have evaluated iron indices across different brain regions in RLS (Haba-Rubio et al. 2005; Astrakas et al. 2008; Godau et al. 2008; Margariti et al. 2012; Rizzo et al. 2013; Moon et al. 2015; Li et al. 2016; Stefani et al. 2019), including SN, red nucleus, putamen, globus pallidus, caudate, thalamus, pons, and dentate nucleus. There is substantial heterogeneity in the findings from these studies, which use different techniques (Beliveau et al. 2022). The overall evidence suggests impaired brain iron homeostasis in RLS, particularly in the SN (Earley et al. 2022). Importantly, brain iron concentrations measured by MRI increased in the SN and prefrontal cortex 2 weeks after IV iron therapy in participants with RLS (Earley et al. 2004). More recently, significant changes in susceptibility values on MRI, which may better reflect iron levels, were observed in the caudate nucleus, putamen, and pulvinar thalamus in RLS patients after IV iron therapy, with changes at pulvinar and caudate correlated with symptomatic improvement in RLS at six weeks (Kim et al. 2023). Together, these results suggest that the pathogenesis of and treatment response to IV iron in RLS may involve a sensorimotor network spanning different brain regions, including thalamic and dopaminergic pathways (Kocar et al. 2020). Of note, MRI-based methods primarily reflect ferritin-sequestered iron (Vymazal et al. 1995) and cannot distinguish between iron associated with H- or L-ferritin. Therefore, MRI-derived iron metrics may be less sensitive to changes in H-ferritin-associated iron, which may be more important in the development of BID in RLS (Connor et al. 2003).

A series of studies have also used transcranial ultrasound (TCUS) to evaluate brain iron metabolism in RLS, as changes in the echogenicity of the SN have been correlated with its iron content (Zecca et al. 2005). Patients with RLS were found to have SN hypoechogenicity compared with controls, likely reflecting reduced iron content in the SN (Schmidauer et al. 2005; Godau et al. 2007). In addition, the substantia nigra echogenicity index (SNEI) by TCUS was not correlated with multiple serum iron parameters, suggesting a divergence between serum iron parameters and iron content in SN (Garcia-Malo et al. 2021). However, SNEI increased after IV iron treatment in patients with RLS, more so in those with severe hypoechogenic SN, which also correlated with a greater reduction in their need for dopaminergic medications (Garcia-Malo et al. 2020a, b). This finding highlighted SNEI as a potential predictor of therapeutic response to iron therapy in RLS (Garcia-Malo et al. 2020a, b).

### Neuropathological and laboratory evaluations of brain iron deficiency in RLS

Neuropathological studies provided strong evidence for altered brain iron metabolism in RLS. Connor et al. showed markedly decreased iron staining in the neuromelanin cells in the SN. Consistent with this reduction in iron staining in the SN, histological antibody-determined staining in neuromelanin cells or protein concentration from western blots showed relative decreases in H-ferritin, DMT1, ferroportin, and transferrin receptor (TfR) and an increase in transferrin in SN in RLS (Connor et al. 2003, 2004). In contrast, the relative concentration of L-ferritin in SN in RLS is similar between RLS and control patients. On histological assessment, however, L-ferritin is disproportionately found in astrocytic-type cells (astrocytes/microglia), compared to oligodendrocytes, where the majority of L-ferritin is commonly found. There were no histological findings to suggest microgliosis in the RLS brain (Connor et al. 2003). This shift may indicate an alteration in iron homeostatic signaling in astrocytes that compose the blood-brain barrier, leading to an aberrant failure to redistribute iron in the RLS brain (Connor et al. 2003). Beyond impaired iron acquisition, cellular iron insufficiency may also result from redistribution of iron to mitochondria in neuromelanin cells, reflecting a secondary response to metabolic insufficiency in these cells in RLS (Snyder et al. 2009). Evidence of abnormal iron regulation has also been found outside of SN, including the putamen and at the blood-brain interface, including the choroid plexus and microvessels (Clardy et al. 2006b; Connor et al. 2011). BID may stem from impaired iron transport across the blood-brain barrier, as reflected by decreased iron uptake and storage within endothelial cells at brain microvessels (Connor et al. 2011). Taken together, these studies provided evidence for cellular iron insufficiency in SN and other brain regions, which may arise from a variety of mechanisms, including reduced iron acquisition by neurons and/or reduced iron transport across the blood-brain barrier.

Dysregulation of iron export may also contribute to BID in RLS. For instance, the levels of total ferritin in serum-derived neuronal extracellular vesicles (nEV) from individuals with RLS were higher than those in controls. With further analysis, H-ferritin, but not L-ferritin, was the primary component increased in these vesicles in RLS compared to control subjects (Chawla et al. 2019). In women with iron-deficiency anemia (hemoglobin < 11 g/l), the serum-derived nEVs fromwomen with RLS showed a significant increase in ferritin and ferroportin and a decrease in TfR compared with women without RLS (Manolopoulos et al. 2024). Despite severe systemic iron deficiency, the RLS subjects still excreted a disproportionately greater amount of ferritin. Additionally, increased ferroportin (iron exporter) and increased DMT1 and TfR (iron importers) expression have been reported in the lymphocytes from females with early-onset RLS. There were no differences, however, in H- or L-ferritin levels in lymphocytes between RLS and control subjects, suggesting increased iron turnover rather than iron depletion in these cells in individuals with RLS (Earley et al. 2008). It was also suggested that the primary pathology may be the increase in the iron exporter (ferroportin) with secondary compensation by increasing iron import (DMT1 and TfR) (Earley et al. 2008). In studies assessing the long-term rate of iron elimination in RLS patients following a standard IV iron infusion, it appears that in RLS patients the loss of iron, as determined by changes in serum ferritin, occurs at a rate 2–11 times greater than predicted from historical data from the general population (Earley et al. 2004). The studies on increased ferritin in nEVs, changes in iron metabolism in RLS lymphocytes, and the increased iron loss in RLS patients support the hypothesis that one component of the altered iron homeostasis in RLS is increased cellular/organ/body iron excretion.

### Brain iron deficiency and neurotransmitter dysfunction in RLS

Brain iron deficiency has been closely linked to dopaminergic dysfunction. The evidence pointing to this association has been comprehensively reviewed previously (Earley et al. 2014). Only a brief overview was provided to facilitate the understanding of the biological consequences of BID in RLS. BID in animal models showed increased levels of tyrosine hydroxylase (TH) and phosphorylated TH (pTH), the rate-limiting enzyme for dopamine synthesis, in the basal ganglia. This was consistent with similar findings from studies using postmortem tissues from RLS patients (Connor et al. 2009). This increase in TH and pTH expression and activity has been attributed at least in part to the upregulation of the hypoxia-inducible factor (HIF) pathway, contributing to increased dopamine synthesis (Patton et al. 2011). BID was also associated with decreased membrane-bound dopamine reuptake transporter (DAT) and increased extracellular dopamine in animal models (Nelson et al. 1997; Bianco et al. 2008). Consistently, imaging studies in RLS patients have shown a possible decrease in membrane-bound DAT (Earley et al. 2011; Lin et al. 2016). Together, these findings support a presynaptic hyperdopaminergic state. In contrast, at the level of dopamine receptors, studies suggest impaired dopaminergic signaling. RLS patients had lower D2 receptor (D2R) in the putamen and a lower D2 receptor binding potential (Earley et al. 2014). Notably, the efficacy of dopamine agonists used to treat RLS is thought to be primarily mediated by the short isoform of the D2R in the dopaminergic neurons and D4R in the glutamatergic terminals, although the precise receptor mechanisms underlying symptom relief remain incompletely understood (Ferré et al. 2026).

Brain iron deficiency has also been linked to dysfunction in the glutamatergic system. For instance, BID rats demonstrated hypersensitive corticostriatal glutamatergic terminals, as evidenced by optogenetically induced glutamate release at a lower frequency, which was significantly counteracted by locally perfused pramipexol, ropinirole, and gabapentin, medications used to treat RLS (Yepes et al. 2017). Consistently, RLS patients showed increased Glx peaks in the proton magnetic resonance spectroscopy (MRS) (Allen et al. 2013b), and transcranial magnetic stimulation studies in RLS patients have demonstrated a hyperactive motor cortex, which could be attributed to increased glutamatergic activity (Tergau et al. 1999). These results suggest hypersensitivity in the corticostriatal glutamatergic terminal in the presence of brain iron deficiency could be an important pathogenic mechanism in RLS.

The adenosine system has also been evaluated in the BID models of RLS, given that adenosine is a strong sleep-promoting neurotransmitter and a potent regulator of dopaminergic and glutamatergic neurotransmission (Ferré 2019). Indeed, BID rodents showed downregulation of adenosine A1 receptor in the cortex and striatum, which could contribute to the hyperarousal phenomenon in RLS (Quiroz et al. 2016). However, supporting evidence for this observation from human studies is yet to be established (Ferré 2019).

### Genes linked to iron metabolism and RLS

A number of genes were implicated in RLS through genome-wide association studies (Stefansson et al. 2007; Winkelmann et al. 2007). Among them, some have been linked to iron homeostasis, including BTBD9, MESI1, and possibly PTPRD, although none of them is a classic iron regulatory gene. In an Icelandic sample, a common BTBD9 variant was associated with RLS and periodic limb movements in sleep in a genome-wide association study. This variant is not specific to RLS, as it is also associated with periodic limb movements in sleep without RLS. Each risk allele is associated with 13% lower serum ferritin levels (Stefansson et al. 2007). Similarly, a frequent polymorphism in BTBD9 was significantly associated with serum ferritin levels in a study involving Danish blood donors (Sørensen et al. 2012). The BTBD9 variants have also been linked to higher hepcidin levels (Fawzy et al. 2021). In BXD mouse models, striatal expression of mouse *btbd9* has shown a positive correlation with iron concentration in the ventral midbrain (Jellen et al. 2009). Together, these observations suggest that BTBD9 influences both central and peripheral iron concentrations.

MEIS1 is another RLS-predisposing gene identified through a large genome-wide association study (Winkelmann et al. 2007). It encodes a transcription factor involved in hematopoiesis and neural crest development in embryonic tissues (Azcoitia et al. 2005; Maeda et al. 2001). Its exact function in postembryonic cells is poorly understood. In RLS, MEIS1 mRNA expression is decreased in brain tissues from individuals who are homozygous carriers of an intronic RLS risk haplotype (Xiong et al. 2009). Silencing of the MEIS1 mRNA expression in a lymphoblastoid cell line resulted in an increase in BTBD9, transferrin-2 receptor, and ferroportin mRNA expression and a decrease in hepcidin mRNA expression. These observations suggest a possible role for MEIS1 in regulating diverse pathways in cellular iron metabolism, such as mitochondrial iron transport, cellular iron export, and BTBD9 expression (Earley et al. 2014). Further, iron status has been shown to regulate MEIS1 expression in vitro (Wade, Chiou, and Connor 2019). Iron chelation increased MEIS1 expression, whereas iron loading decreased MEIS1 expression in a cell culture model of the blood-brain barrier (Connor et al. 2017). This finding is consistent with increased MEIS1 levels in the microvasculature in the RLS brain (Connor et al. 2017). In contrast, MEIS1 expression was decreased under iron-deficient conditions and was not affected by iron loading in HeLa and neuroblastoma cell lines (Catoire et al. 2011). Although these findings require further validation, they suggest that MEIS1 expression may be differentially regulated by iron status in neural and non-neural cell types. Overall, additional study is needed to further clarify the link between MEIS1, iron metabolism, and RLS.

PTPRD is a member of the type IIa receptor-like protein tyrosine phosphatases and has functions in motor neuron axon targeting during embryonic development (Schormair et al. 2008). Genetic knockout of this gene in mice leads to alterations in locomotor activity and sleep, though the underlying mechanism remains unclear (Drgonova et al. 2015). Of note, a systems-genetics analysis in BXD mice identified PTPRD as a candidate modifier of the systemic response to dietary iron deficiency (Yin et al. 2012); however, whether PTPRD variants influence iron homeostasis in patients with RLS remains unknown.

### Iron therapy for RLS

Norlander first reported the benefits of IV iron treatment for RLS in patients with and without anemia in 1953 (Nordlander 1953). Since then, numerous clinical trials and studies have evaluated the effectiveness of iron therapy for RLS. Broadly, they can be divided into randomized placebo-controlled trials and open-label case series, which we will examine separately.

### Randomized placebo- controlled trials evaluating the effectiveness of iron therapy in RLS

Numerous randomized placebo-controlled randomized clinical trials (RCTs) have been reported between 2000 and 2024 evaluating iron therapy in RLS. Iron therapies that have been studied include oral iron therapy, IV iron dextran, IV iron sucrose, IV ferric carboxymaltose (FCM), and IV ferumoxytol. Regarding oral iron therapy, two RCTs (Davis et al. 2000; Wang et al. 2009) tested ferrous sulfate 325 mg (60 mg elemental iron) twice daily for 12 weeks. Wang et al., who enrolled participants with lower ferritin levels (15–75 µg/L), found significant symptom improvement with iron therapy (Wang et al. 2009). In contrast, Davis et al., who included participants with a wider ferritin range (9–690 µg/L; mean 134.8 µg/L), found no benefit of oral iron therapy for symptoms or sleep (Davis et al. 2000). Another study also compared the efficacy of oral iron with pramipexol in improving RLS symptoms in patients with low ferritin (15–50 ug/L) and reported no difference between these two therapies in improving RLS symptoms (Lee et al. 2014). Together, these findings suggest oral iron is likely more effective in RLS patients with lower ferritin levels. Current AASM guidelines recommend oral iron therapy for adult RLS patients with ferritin ≤ 75 µg/L (Winkelman et al. 2025).

Among IV iron therapies in RLS, FCM has the strongest evidence base. Six RCTs employed FCM at 500–1500 mg and demonstrated the efficacy and safety of FCM in improving RLS symptoms in patients with or without IDA at 4–12 weeks after infusion (Allen et al. 2011; Cho et al. 2016; Trenkwalder et al. 2017; Bae et al. 2021a; Earley et al. 2024), except that a single dose of 500 mg FCM did not show significant benefits (Cho et al. 2018). A few studies with longer follow-up showed that the benefits of FCM could last 6 months to over a year (Allen et al. 2011; Cho et al. 2016; Bae et al. 2021a). Participants with lower transferrin saturation (TSAT) values appear to have greater symptomatic improvement after FCM infusion (Trenkwalder et al. 2017). Additionally, FCM was shown to be more helpful in patients with RLS without augmentation than in those with augmentation (Earley et al. 2024). Other IV iron therapies that have been studied include iron dextran and iron sucrose. One double-blinded RCT showed that 1000 mg of iron dextran resulted in significant but transient improvement in RLS in patients with RLS and end-stage renal disease (Sloand et al. 2004). Similarly, one RCT showed that iron sucrose at 1000 mg significantly improved RLS symptoms in hemodialysis patients (Deng et al. 2017). Two RCTs also evaluated the efficacy of iron sucrose in the treatment of idiopathic RLS, with one study showing no convincing benefits of iron sucrose over placebo after 2 weeks of treatment (Earley et al. 2009) and the other showing some evidence for improvement in the acute phase and long-term follow-up (Grote et al. 2009). One reason iron sucrose may be inferior to FCM in efficacy is that it is a fast-release iron product. Slow-release iron formulations such as FCM may allow more iron to be taken up by macrophages and made available for uptake by the brain (Allen et al. 2018). Overall, these studies have led to a strong recommendation for FCM in the treatment of RLS in a clinical guideline from AASM (Winkelman et al. 2025). Future studies are needed to evaluate whether other IV iron formulations have comparable efficacy to FCM and whether IV iron therapy is effective in RLS patients with augmentation.

### Open-label case series of iron therapy in RLS

Open-label case series studies provided additional insights into iron therapy for RLS. These included evaluations of different iron formulations, such as iron dextran (Earley et al. 2004; Cho et al. 2013; Mehmood et al. 2014), FCM (Adenekan et al. 2013; Schneider et al. 2015; Lieske et al. 2016; Bae et al. 2023), and iron sucrose (Birgegård et al. 2010; Grim et al. 2013; Zhang et al. 2015). Overall, these studies provided support for IV iron therapy for RLS across various cohorts, although iron sucrose was deemed less effective than FCM or low-molecular-weight (LMW) iron dextran (Allen et al. 2018). One study assessed repeated iron treatment in a small cohort of RLS patients and found it helpful for sustaining improvement in RLS symptoms in those with a faster decline in ferritin levels (Earley et al. 2005b). Another study evaluated the risk of iron overload and anaphylaxis following FCM treatment of RLS in real-world clinical conditions and found the risk to be low (Garcia-Malo et al. 2020a), which is consistent with findings from other studies (Allen et al. 2018). A retrospective study assessed predictors of response to FCM treatment and found that lower transferrin saturation was associated with better response, especially in female patients with RLS (Park et al. 2020). While these studies provided valuable information on IV iron therapies for RLS, most findings need to be validated in larger, well-designed prospective studies.

## Conclusion

Based on a synthesis of the current literature, the following conclusions can be drawn about altered iron metabolism and RLS. Peripheral iron deficiency is closely linked to RLS and is a major trigger of its development, whereas BID has been recognized as a core mechanism in its pathogenesis. Peripheral iron indices primarily reflect the body’s iron stores for erythropoiesis and are poorly correlated with brain iron status. Regarding BID in RLS, existing studies, particularly neuropathological studies, suggest impaired iron uptake or transport at the blood-brain barrier and in different brain regions of the nigrostriatal system, leading to cellular iron insufficiency. Indeed, decreased iron content in the SN has been the most consistent finding, supported by numerous neuroimaging studies. However, there is substantial heterogeneity in the literature, with some studies showing normal or increased iron content in subcortical brain regions in RLS. Additionally, altered iron export in the brain or other peripheral tissues/cells may contribute to iron dysregulation in RLS. Altogether, the current model centers on BID, which leads to disturbances in dopaminergic and other related pathways, triggering the sensorimotor symptoms of RLS (Ferré et al. 2019).

Iron therapy has been recognized as a component of the standard of care for the treatment of RLS, including both oral and IV iron therapy. Unlike dopamine agonists and α2δ ligands, which primarily provide symptomatic relief by modulating downstream neurotransmission, iron therapy aims to correct the presumed upstream brain iron deficiency. By restoring brain iron homeostasis, iron treatment may influence multiple downstream mechanisms implicated in RLS, including dopaminergic, glutamatergic, and adenosinergic signaling. Future studies are needed to elucidate the precise mechanisms underlying BID and the pathogenesis of RLS, identify more accurate prognostic biomarkers of iron therapy in RLS, and determine the comparative efficacy and optimal use of different oral and IV iron therapies in RLS.

## Data Availability

No datasets were generated or analysed during the current study.
